# Editorial

**Published:** 1915-02

**Authors:** 


### EDITORIAL

The Business Office of the *Journal-Record* is 23 West Alabama Street.
The Editorial Office is 1014-15 Atlanta National Bank Building.
Address all Business Communications to *Journal-Record of Medicine*, 23 West Alabama Street.
Make remittances payable to *The Journal-Record of Medicine*.
On matters pertaining to the Editorial and Original Communications, address Edgar G. Ballenger, M. D., Atlanta, Ga.
Reprints of Original Articles will be furnished at cost price. Requests for the same should always be made in THE MANUSCRIPT.
We will present, postpaid, on request to each contributor of an original article, twenty (20) marked copies of *The Journal-Record of Medicine* containing such article.

### OPIATES AND NARCOTICS.

The Federal law controlling the sale of opiates and all cocaine products goes into effect on March 1 and it is to be highly commended. Nothing in the history of legislation as to drugs has its dignity and importance. We are opposed as a rule to all sumptuary legislation, but this has character that goes far beyond an attempt to make people moral by law.

In the first place, the evils it aims to eliminate are well known and so great as to be a menace to our civilization, and in the second place, the law does not attempt to limit in the slightest degree the legitimate practice of medicine or to take from the physician the control and care of the patient who needs his assistance as habitues in the use of drugs.

There is just a little ambiguity about the wording of the law because lawyers have not yet learned that a thing can be well said and simply said at the same time. The obscurity of expression comprises the control of the two ways in which a physician may use the drugs in question. The first way, that of prescribing for his patients, needs only that the prescription be clearly written and fully signed and marked with the doctor's license number. Any blank may be used. The other way covers the purchase by the doctor, himself, of drugs that he plans to use in his rounds or to dispense. A prescription for this purpose must be written upon a special blank provided by the Internal Revenue Department.

The ultimate results of the law can not be other than good if it receives the support of the Profession it is entitled to. No prescription can be refilled; no man can tell his neighbors what a good medicine he has had from a doctor and advise them to get some of the same. Neighborhood prescribing with its consequent ills will cease so far as opiates are concerned, and refills for the case originally attended will not be permitted to go far toward establishing a habit. Responsibility is placed where it belongs by the law and better than that, it is kept there. No longer can a patient say that a certain doctor gave an opiate and thus started the habit, when as a matter of fact, only one small prescription was written.

A great problem will arise as to the care of habitues. Many of them must have some measure of their drugs or die and it will be the unpleasant duty of the doctors to care for them. But this will be faced and as it is met there will be the assurance that the patient can not get the drug surreptitiously when the treatment is about to be successful.

That there may be men so far degraded as to use their authority in prescription writing to pander to the drug users, is probably true, but these very men will have to give an accounting of themselves and their ways sometime to Federal authorities. They will be driven from their methods and the law will have accomplished another part of its purpose.

Inconveniences will arise at first in the use of prescriptions, but they will be so slight as to disappear as soon as one is familiar with the law. The beneficial results will at once offset them.

R. R. D.

---

To the Alumni of the University of Maryland: Dr. T. A. Ashby, of the University of Maryland, is organizing an Alumni Association of the University of Maryland for the State of Georgia. There are many graduates from this well-known institution in Georgia. Dr. Ashby requests that as many of the alumni as possible attend the Macon meeting of the Medical Association of Georgia and that they organize at this time a

State Alumni Association. Show your loyalty to your venerable and esteemed alma mater by attending this meeting and become one of the charter members of this proposed association.

